# Valorization of cassava-based agro-industrial byproducts as ensiled total mixed fiber for sustainable roughage replacement in early-lactating dairy cows

**DOI:** 10.14202/vetworld.2026.3268-3281

**Published:** 2026-07-28

**Authors:** Nawanon Chantaprasarn, Wiriya Loongyai, Sornthep Tumwasorn, Phongthorn Kongmun

**Affiliations:** 1 Department of Animal Science, Faculty of Agriculture, Kasetsart University, Bangkok 10900, Thailand.

**Keywords:** agro-industrial byproducts, cassava bioethanol waste, cassava pulp, dairy cows, ensiled total mixed fiber, milk production, rumen fermentation, sustainable livestock production

## Abstract

**Background and Aim:**

Sustainable utilization of agro-industrial byproducts offers an opportunity to reduce feed costs and dependence on conventional forages in tropical dairy production. Cassava pulp and cassava bioethanol waste are abundant residues with potential as alternative roughage sources, yet their application as ensiled total mixed fiber (TMF) in early-lactating dairy cows has not been systematically evaluated. This study investigated the effects of ensiled TMF formulated with cassava pulp or cassava bioethanol waste as complete roughage replacements for guinea grass on feed intake, nutrient digestibility, rumen fermentation, blood metabolites, milk yield, and milk composition in early-lactating dairy cows.

**Materials and Methods:**

Eighteen multiparous Holstein Friesian crossbred cows (25 ± 15 days in milk) were allocated to three dietary treatments in a randomized complete block design (n = 6/group): fresh guinea grass (control), ensiled TMF containing cassava pulp (TMFc), or ensiled TMF containing cassava bioethanol waste (TMFe). All diets were offered *ad libitum* with a roughage-to-concentrate ratio of 40:60 over a 90-day feeding period. Feed intake, apparent nutrient digestibility, rumen fermentation characteristics, blood urea nitrogen, milk yield, and milk composition were determined.

**Results:**

Cows fed TMFe showed significantly greater dry matter intake (15.6 ± 0.61 kg/day), nutrient intake, and metabolizable energy intake (36.10 ± 1.26 Mcal/day) than those receiving guinea grass (p < 0.01). Apparent digestibility of dry matter, organic matter, crude protein, neutral detergent fiber, and acid detergent fiber remained unchanged among treatments (p > 0.05). Ruminal pH and temperature were maintained within physiological ranges. At 4 h post-feeding, TMFc increased blood urea nitrogen (21.2 ± 1.89 mg/dL; p = 0.047), ruminal ammonia nitrogen (14.24 ± 0.35 mg%; p = 0.001), and butyrate production, whereas the control diet produced higher propionate concentrations (p = 0.044). Milk composition was unaffected by dietary treatment, while TMFc showed a tendency toward greater milk yield (15.5 ± 0.82 kg/day; p = 0.069).

**Conclusion:**

Ensiled TMF formulated from cassava-based agro-industrial byproducts can effectively replace conventional guinea grass without compromising nutrient digestibility, rumen stability, or milk composition in early-lactating dairy cows. Cassava bioethanol waste-based TMF improved voluntary feed intake and energy supply, whereas cassava pulp-based TMF enhanced ruminal nitrogen metabolism and tended to increase milk yield. These findings demonstrate the potential of cassava-derived byproducts as sustainable alternative roughage resources that support circular bioeconomy principles and improve feed resource utilization in tropical dairy production systems.

## INTRODUCTION

Roughage is essential for maintaining rumen function, animal health, and fermentation efficiency in dairy cows. Its fibrous structure promotes rumen stratification, microbial colonization, and saliva secretion, which buffers ruminal pH and helps prevent acidosis [1]. However, although roughage is indispensable, its inclusion must be balanced with energy-rich dietary components to avoid excessive dilution of dietary energy, particularly in high-producing dairy cows [2]. In tropical production systems, rice straw is widely used because of its abundance, but its low crude protein (CP) content and high lignin concentration restrict digestibility and reduce nutrient availability for rumen microorganisms. Although physical, chemical, and biological treatments have been developed to improve its nutritive value, their adoption remains limited because of economic and logistical constraints in smallholder farming systems. Previous *in vivo* studies in small ruminants have demonstrated that monosodium glutamate by-product-treated rice straw can serve as an alternative roughage source by improving growth performance and rumen fermentation at moderate inclusion levels, whereas excessive substitution may negatively affect feed intake and digestibility [3]. Furthermore, tropical forage resources generally contain low CP and high structural fiber, particularly during the dry season when forage quality deteriorates further. These nutritional limitations constrain microbial protein synthesis, reduce feed utilization efficiency, and ultimately impair animal productivity [4, 5]. Although forage legumes and nitrogen supplementation can partially alleviate these deficiencies, there remains a need for alternative roughage resources that are nutritionally superior, economically feasible, and readily available.

The continued expansion of cassava-processing industries has generated substantial quantities of agro-industrial byproducts, including cassava pulp, cassava peels, cassava bagasse, and cassava bioethanol waste. Improper disposal of these residues may contribute to environmental pollution, whereas their utilization as feedstocks for biofuel production, biodegradable materials, activated carbon, and animal feed provides opportunities for value addition and sustainable resource utilization [6–10]. Approximately 2.5 tons of cassava bagasse and 100–300 kg of cassava peel are generated for every ton of cassava starch produced, highlighting the considerable potential of these byproducts for beneficial utilization. Nutritionally, cassava-derived residues are rich in fermentable carbohydrates while providing moderate concentrations of CP and functional fiber. Cassava roots contain 81.0–87.1 g/100 g of carbohydrates, whereas cassava leaves contain 21.2–28.4 g/100 g of protein and 16.1–22.9 g/100 g of fiber [11]. In addition, cassava pulp can be processed into dietary fiber containing up to 89.2% total fiber [12]. Anti-nutritional compounds, including cyanogenic compounds, can be effectively reduced through boiling, drying, or microbial fermentation, thereby improving the safety and feeding value of cassava-derived products for livestock [11].

Total mixed fiber (TMF) is a feeding strategy that combines multiple fibrous feed ingredients, frequently including agro-industrial byproducts, into a homogeneous ration to optimize nutrient supply in ruminant diets. TMF promotes uniform feed intake, minimizes feed sorting, and improves nutrient utilization, thereby supporting rumen health and animal performance. Previous studies demonstrated that TMF improved the digestibility of dry matter (DM), organic matter (OM), and CP, resulting in greater energy intake and increased milk production in mid-lactating dairy cows compared with conventional roughage sources [13]. Ensiling TMF with sodium diacetate further improves its nutritional quality by increasing DM and OM concentrations during storage [14]. Further-more, modification of additive type and inclusion level enables optimization of fiber quality and feed preservation. TMF also positively influences rumen fermentation. Diets with lower non-fiber carbohydrate-to-neutral detergent fiber (NDF) ratios improve growth performance and nutrient digestion, whereas excessive non-fiber carbohydrate may impair ruminal function [15]. Likewise, organic mineral supplementation in TMF has been shown to improve rumen fermentation and animal performance in beef cattle [16]. An important characteristic of TMF is its peNDF, which stimulates chewing activity and saliva secretion, thereby maintaining ruminal pH and improving fiber digestibility [17]. Higher peNDF concentrations may also modify milk fatty acid composition and consequently influence milk quality [18]. Moreover, incorporating agro-industrial byproducts into TMF further enhances its nutritional and economic value. Alkali-treated sugarcane bagasse has been reported to improve milk production and body weight (BW) gain, whereas cassava pulp supplemented with yeast waste can replace up to 75% of soybean meal without adversely affecting rumen fermentation [19]. In addition, fermentation and enzymatic bioprocessing techniques further improve protein quality and fiber digestibility of these feed resources [20].

Despite increasing interest in cassava-derived by-products as alternative feed resources for ruminants, important knowledge gaps remain regarding their application as primary roughage sources in ensiled TMF systems, particularly during early lactation. Most previous studies have evaluated TMF in mid-lactating dairy cows or investigated cassava byproducts as feed supplements, protein sources, or concentrate ingredients rather than as complete roughage replacements. Furthermore, available studies have primarily focused on dried or yeast-fermented cassava byproducts, whereas information on ensiled TMF formulated from different cassava-derived residues remains scarce [13, 21]. Consequently, the comparative effects of cassava pulp- and cassava bioethanol waste-based TMF on voluntary feed intake, nutrient digestibility, ruminal fermentation, and lactational performance have not been systematically investigated under early-lactation conditions, when nutrient demands are greatest and cows are particularly susceptible to negative energy balance. Addressing these knowledge gaps is essential to establish scientifically validated, sustainable, and economically viable roughage alternatives for tropical dairy production systems while promoting circular bioeconomy principles through the valorization of cassava-processing residues.

Therefore, this study aimed to evaluate the effects of ensiled TMF formulated with cassava pulp (TMFc) or cassava bioethanol waste (TMFe) as complete replacements for guinea grass on feed intake, apparent nutrient digestibility, ruminal fermentation characteristics, and lactational performance in early-lactating dairy cows. It was hypothesized that cassava-based ensiled TMF could effectively replace conventional guinea grass without compromising nutrient utilization, rumen function, milk yield, or milk composition while providing new comparative evidence on the feeding value of two distinct cassava-derived agro-industrial byproducts and supporting the development of sustainable feeding strategies for tropical dairy production systems.

## MATERIALS AND METHODS

### Ethical approval

This study was conducted as part of a Ph.D. research project initiated in 2015 at the Dairy Farming Promotion Organization of Thailand, Muak Lek, Saraburi Province, Thailand. The experimental protocol was reviewed and approved by the Thesis Proposal Committee of the Graduate School, Kasetsart University, Thailand. The animal experiment was performed in 2016, before the establishment of the Institutional Animal Care and Use Committee (IACUC) at the institution; therefore, an IACUC approval number was not available. Nevertheless, all animal handling, management, and sample collection procedures were performed by trained personnel in accordance with accepted animal welfare standards for dairy cattle and institutional guidelines applicable at the time of the study. Throughout the experimental period, the animals were maintained under routine farm management conditions, provided free access to clean drinking water, and monitored daily to ensure their health and well-being.

### Study period and location

The study was conducted from January to April 2016 at the Dairy Farming Promotion Organization of Thailand, Muak Lek District, Saraburi Province, Thailand.

### Study design

The feeding trial was conducted at the Dairy Farming Promotion Organization of Thailand, Muak Lek District, Saraburi Province, Thailand. Eighteen multiparous Holstein Friesian crossbred cows averaging 25 ± 15 days in milk and an initial BW of 429 ± 29 kg were enrolled in the study. The animals were blocked according to parity and days in milk and randomly allocated within each block to one of three dietary treatments using a randomized complete block design (RCBD), with six cows per treatment (n = 6). Each cow was housed individually in a well-ventilated pen equipped with a permanent roof and had unrestricted access to clean drinking water and mineral supplements.

Three dietary treatments were evaluated. The control group received freshly harvested guinea grass, whereas the two experimental groups received ensiled TMFc or ensiled TMFe. Unlike previous studies that evaluated cassava-derived byproducts as feed supplements, protein sources, or concentrate ingredients, the present study investigated their use as primary roughage sources within ensiled TMF systems.

The guinea grass used in the control treatment was harvested and offered fresh daily. In contrast, the TMFc and TMFe diets were thoroughly mixed, ensiled under anaerobic conditions for 7 days to facilitate lactic acid fermentation, and subsequently stored in 25-kg plastic bags until feeding. During storage, the ensiled diets were routinely inspected, and no visible mold growth, abnormal odor, or signs of spoilage were observed, indicating acceptable preservation quality.

Although peNDF was not quantified, the experimental diets were formulated to provide adequate physically effective fiber based on the physical characteristics and particle size of the roughage ingredients. Both guinea grass and the ensiled TMF diets contained sufficient structural fiber to stimulate chewing activity and saliva secretion.

All cows received their assigned roughage *ad libitum* and were supplemented with a concentrate containing 20% CP. Total feed allowance was calculated at 3.5% of BW while maintaining a roughage-to-concentrate ratio of 40:60. Before data collection, all animals underwent a 14-day adaptation period to acclimate to the experimental diets and management conditions.

Roughage was offered separately from the concentrate twice daily on an *ad libitum* basis. Feed allowances were adjusted daily to maintain approximately 10% refusals and ensure unrestricted voluntary intake. Concentrate was offered individually during the morning (06:00 h) and evening (16:00 h) milking sessions.

The concentrate was obtained from a commercial feed manufacturer routinely supplying the Dairy Farming Promotion Organization of Thailand during the experimental period. Because the formulation was proprietary, detailed ingredient composition was unavailable. Nevertheless, the chemical composition of the concentrate, including DM, CP, EE, ash, NDF, and acid detergent fiber (ADF), was determined by laboratory analysis and is presented in Table 1.

Feed intake and milk yield were recorded throughout the 90-day feeding period. Nutrient requirements and feed intake were estimated according to NRC [22]. Although the diets were not formulated to be strictly isocaloric or isonitrogenous, all treatments were designed to meet or exceed the minimum nutrient requirements recommended by NRC [22] for early-lactating dairy cows. Based on the observed milk yield and stage of lactation, the nutrient composition of all diets was considered adequate to support maintenance and milk production under the conditions of the present study.

### Feed intake and BW measurement

Daily feed intake was calculated as the difference between the amount of feed offered and the refusals recorded for each cow. BW was measured individually at the beginning and end of the experiment using a calibrated livestock digital scale to monitor BW changes and ensure accurate adjustment of feed allowances.

### Feed and fecal sampling and chemical analysis

Representative samples of feed and feed refusals were collected throughout the experimental period for chemical analyses. Fecal samples were collected directly from the rectum by grab sampling during the final 7 consecutive days of the feeding trial. Samples were pooled by animal, dried at 60°C for 72 h, ground to pass through a 1-mm screen, and analyzed for acid-insoluble ash (AIA).

Apparent nutrient digestibility was estimated using AIA as an internal marker according to Van Keulen and Young [23] using the following equation:

Apparent digestibility (%) = 100 − [100 × (% AIA in feed / % AIA in feces) × (% nutrient in feces / % nutrient in feed)]

where AIA represents the acid-insoluble ash concentration (% DM basis) and nutrient represents the concentration of the respective nutrient in feed and feces.

DM, CP, EE, and total ash were analyzed according to AOAC International [24]. NDF, ADF, and acid detergent lignin (ADL) were determined using the method described by Van Soest *et al.* [25].

### Rumen fluid and blood sampling

On the final day of the experiment, rumen fluid samples were collected using a stomach tube immediately before the morning feeding (0 h) and 4 h after feeding to determine rumen fermentation characteristics. To minimize saliva contamination, the initial portion of rumen fluid was discarded before sample collection. Ruminal pH was measured immediately using a digital pH meter. The rumen fluid was subsequently filtered through four layers of cheesecloth and centrifuged at 16,000 × *g* for 15 min. The resulting supernatant was stored at −20°C until determination of NH₃–N and volatile fatty acid (VFA) concentrations.

Ammonia nitrogen concentration was determined using the colorimetric method described by Chaney and Marbach [26]. Individual VFA concentrations were quantified by high-performance liquid chromatography using a Waters 600E system equipped with a Waters 484 ultraviolet detector and a Novapak C18 column (3.9 × 300 mm) (Waters Corporation, Milford, MA, USA). The mobile phase consisted of 10 mmol/L H₂PO₄ adjusted to pH 2.5 according to the method of Samuel *et al.* [27].

Simultaneously, blood samples were collected from the jugular vein at 0 and 4 h after feeding. Samples were immediately placed on ice, maintained at 4°C for 1 h, and centrifuged at 3,500 × *g* for 20 min to obtain plasma. Plasma samples were stored at −20°C until blood urea nitrogen (BUN) analysis according to Crocker [28].

### Milk yield and composition

Milk yield was recorded individually at each milking session conducted twice daily. Milk samples were collected before the start of the experiment and subsequently at 2-week intervals. On each sampling day, milk from both morning and afternoon milking sessions was thoroughly mixed, and approximately 100 mL was collected for analysis. Milk composition, including total solids, fat, protein, lactose, and solids-not-fat, was determined using an Electric Milk Tester (FOSS Analytical A/S, Hillerød, Denmark).

### Statistical analysis

Data were analyzed using the general linear model procedure implemented in SAS software (SAS Institute Inc., Cary, NC, USA) [29] according to an RCBD. The statistical model included treatment as a fixed effect and block (parity and days in milk) as a blocking factor. Treatment means were compared using Duncan's multiple range test, and statistical significance was declared at p < 0.05 [30]. Results with 0.05 ≤ p < 0.10 were interpreted as tendencies.

The sample size was determined based on animal availability and practical constraints under field conditions. Because an *a priori* statistical power analysis was not performed, findings showing tendency-level significance, such as milk yield, should be interpreted with appropriate caution.

## RESULTS

### Feed composition

The chemical composition of the experimental roughage sources is presented in Table 1. Guinea grass, used as the control roughage, contained 5.8% CP, 75.4% NDF, and 44.0% ADF on a DM basis. Compared with guinea grass, both ensiled TMF rations, TMFc and TMFe, contained higher CP concentrations (8.9% and 8.8%, respectively) and lower NDF contents (62.1% and 65.9%, respectively). Ensiled TMFe had the highest ADF (46.8%) and ADL content (9.6%), whereas guinea grass and ensiled TMFc had comparable ADL values (7.8% and 8.2%, respectively). The inclusion of agro-industrial byproducts in ensiled TMFc and ensiled TMFe altered the fiber profile and nutritional density of the rations, suggesting a potential advantage in nutrient supply over conventional roughage.

One limitation of the present study is that silage fermentation characteristics, including pH, lactic acid, acetic acid, butyric acid, and NH₃–N concentrations, were not determined during the ensiling process. Therefore, although no visible spoilage, mold growth, or abnormal odor was observed, the fermentation quality of ensiled TMF could not be quantitatively evaluated. Future studies should include comprehensive silage quality assessments to better characterize the fermentation profile and preservation efficiency of cassava-based TMF.

**Table 1:** Feed ingredients and chemical composition of experimental diets containing ensiled total mixed fiber (TMF).

**Items**	**Guinea grass**	**TMFc**	**TMFe**	**Concentrate**
**Amount, % DM**				
Guinea grass	100.0	–	–	–
Bagasse	–	30.0	45.0	–
Vinasse	–	5.0	5.0	–
Urea	–	1.6	1.0	–
Rice straw	–	10.0	10.0	–
Cassava pulp	–	53.4	0.0	–
Cassava bioethanol waste	–	0.0	39.0	–
**Total, kg**	100.0	100.0	100.0	–
**Chemical composition (% DM basis)**				
DM	33.5	26.3	27.0	87.9
CP	5.8	8.9	8.8	21.1
Ash	9.1	7.3	8.3	8.2
EE	1.9	1.0	1.4	3.3
NDF	75.4	62.1	65.9	38.9
ADF	44.0	44.9	46.8	34.5
ADL	7.8	8.2	9.6	9.4

TMFc = ensiled total mixed fiber with cassava pulp; TMFe = ensiled total mixed fiber with cassava bioethanol waste; DM = dry matter; CP = crude protein; EE = ether extract; NDF = neutral detergent fiber; ADF = acid detergent fiber; ADL = acid detergent lignin.

### BW, feed intake, and apparent nutrient digestibility

Animal performance and nutrient utilization data are summarized in Table 2[31]. Dietary treatment had no significant effect on initial BW, final BW, or average daily gain (ADG; p > 0.05). Total DM intake (DMI) was significantly affected by dietary treatment (p = 0.007), with cows fed ensiled TMFe showing the highest intake (15.6 ± 0.61 kg/day), followed by cows fed ensiled TMFc (13.2 ± 0.73 kg/day) and guinea grass (12.6 ± 0.64 kg/day). This study is among the first to demonstrate that ensiled TMF formulated with cassava bioethanol waste can enhance voluntary feed intake and energy supply when used as a primary roughage source in early-lactating dairy cows.

Nutrient intake followed a similar pattern, with significantly higher intakes of DM, OM, NDF, and ADF observed in cows fed ensiled TMFe than in those fed the other treatments (p < 0.001, p < 0.001, p < 0.005, and p < 0.001, respectively). Energy intake, calculated based on digestible OM, was also significantly greater in the ensiled TMFe group (36.10 ± 1.26 Mcal metabolizable energy (ME)/day) than in the ensiled TMFc (30.83 ± 1.43 Mcal ME/day) and guinea grass groups (28.60 ± 1.56 Mcal ME/day; p < 0.001). However, apparent nutrient digestibility did not differ significantly among treatments (p > 0.05). Digestibility coefficients for DM, OM, CP, NDF, and ADF were similar across all groups, indicating that the inclusion of ensiled TMF did not negatively affect digestibility despite higher feed intake.

**Table 2:** Effects of ensiled total mixed fiber (TMF) feeding on body weight gain, feed intake, and apparent nutrient digestibility in early-lactating dairy cows.

**Items**	**Guinea grass**	**TMFc**	**TMFe**	**SEM**	**p-value**
Initial weight, kg	406 ± 19.53	442 ± 34.57	440 ± 32.36	16.21	0.321
Final weight, kg	448 ± 25.38	508 ± 48.48	529 ± 18.90	20.09	0.052
ADG, kg/day	0.58 ± 0.05	0.92 ± 0.28	0.89 ± 0.11	0.10	0.433
Roughage DMI, kg/day	5.4 ± 0.11	6.7 ± 1.00	8.4 ± 0.91	0.53	0.060
% BW	1.3 ± 0.09	1.5 ± 0.30	1.7 ± 0.18	0.12	0.198
g/kg BW⁰·⁷⁵	57.5 ± 3.28	67.7 ± 12.62	79.7 ± 8.47	5.38	0.152
Concentrate DMI, kg/day	7.2 ± 0.58	6.5 ± 0.66	7.2 ± 0.85	0.39	0.625
% BW	1.7 ± 0.14	1.4 ± 0.15	1.5 ± 0.19	0.09	0.443
g/kg BW⁰·⁷⁵	76.8 ± 5.98	64.8 ± 6.74	68.7 ± 8.57	4.06	0.537
**Total DMI, kg/day**	12.6 ± 0.64ᵇ	13.2 ± 0.73ᵇ	15.6 ± 0.61ᵃ	0.50	0.007
% BW	3.0 ± 0.20	2.9 ± 0.34	3.1 ± 0.13	0.14	0.623
g/kg BW⁰·⁷⁵	134.3 ± 7.84	132.5 ± 13.32	148.3 ± 5.58	5.41	0.328
**Nutrient intake, kg/day**					
DM	7.78 ± 0.46ᶜ	8.33 ± 0.32ᵇ	9.77 ± 0.36ᵃ	0.32	<0.001
OM	7.52 ± 0.41ᶜ	8.10 ± 0.38ᵇ	9.53 ± 0.33ᵃ	0.32	<0.001
CP	1.23 ± 0.10	1.26 ± 0.09	1.41 ± 0.21	0.07	0.430
NDF	3.78 ± 0.14ᵇ	3.78 ± 0.27ᵇ	4.68 ± 0.20ᵃ	0.16	<0.005
ADF	2.77 ± 0.13ᶜ	3.01 ± 0.21ᵇ	3.62 ± 0.09ᵃ	0.13	<0.001
Energy intake (Mcal ME/day)	28.60 ± 1.56ᶜ	30.83 ± 1.43ᵇ	36.10 ± 1.26ᵃ	1.20	<0.001
**Apparent nutrient digestibility (%)**					
DM	61.8 ± 1.26	63.5 ± 3.10	61.6 ± 2.69	1.23	0.856
OM	65.5 ± 1.02	67.1 ± 2.88	61.6 ± 2.69	1.28	0.759
CP	66.7 ± 1.10	65.9 ± 4.40	62.1 ± 5.24	1.88	0.975
NDF	55.3 ± 1.22	55.9 ± 2.49	54.8 ± 1.82	0.98	0.983
ADF	57.3 ± 0.80	58.0 ± 1.35	55.2 ± 1.86	0.72	0.873

¹ One kg of digestible OM = 3.8 Mcal ME [31 ]. ᵃ,ᵇ,ᶜ Means in the same row with different superscripts differ significantly (p < 0.05). Values are expressed as mean ± SEM. SEM = standard error of the mean; TMFc = ensiled total mixed fiber with cassava pulp; TMFe = ensiled total mixed fiber with cassava bioethanol waste; ADG = average daily gain; DMI = dry matter intake; BW = body weight; DM = dry matter; OM = organic matter; CP = crude protein; NDF = neutral detergent fiber; ADF = acid detergent fiber; ME = metabolizable energy.

### Rumen fermentation characteristics and blood metabolites

Rumen fermentation characteristics and BUN concentrations are presented in Table 3. Ruminal temperature and pH remained within physiological ranges across all treatments and were not significantly affected by dietary treatment (p > 0.05). Mean ruminal temperature ranged from 38.3 ± 0.54°C to 39.2 ± 0.12°C, whereas mean pH ranged from 6.9 ± 0.09 to 7.1 ± 0.11. At 0 h after feeding, no significant differences were observed among treatments for BUN, NH₃–N, propionate, or acetate concentrations (p > 0.05), indicating that baseline rumen fermentation characteristics were generally comparable among treatments before feeding.

At 4 h after feeding, BUN concentration was highest in cows fed ensiled TMFc (21.2 ± 1.89 mg/dL), which was significantly greater than that in cows fed guinea grass (15.0 ± 1.37 mg/dL; p < 0.05), whereas cows fed ensiled TMFe showed intermediate values. However, mean BUN concentration did not differ significantly among treatments (p = 0.387). The NH₃–N concentration at 4 h after feeding was also highest in the ensiled TMFc group (14.24 ± 0.35 mg%), which was significantly greater than that in the ensiled TMFe and guinea grass groups (p = 0.001), suggesting increased ruminal nitrogen availability with cassava pulp inclusion.

The VFA profile showed no significant differences in mean acetate or butyrate concentrations among treatments. However, mean propionate concentration was significantly higher in the guinea grass group (27.41 ± 1.89 mM) than in the ensiled TMFc (22.36 ± 0.77 mM) and ensiled TMFe groups (22.23 ± 1.67 mM; p = 0.044). Butyrate concentration at 0 h after feeding was significantly higher in cows fed ensiled TMFc than in those fed the other treatments (p = 0.013). Overall, ensiled TMF diets, particularly ensiled TMFc, influenced nitrogen metabolism and selected fermentation end-products without impairing rumen stability.

**Table 3:** Effects of ensiled total mixed fiber (TMF) on rumen fermentation characteristics and blood urea nitrogen (BUN) in early-lactating dairy cows.

**Items**	**Guinea grass**	**TMFc**	**TMFe**	**SEM**	**p-value**
**Temperature, °C**					
0 h post-feeding	37.9 ± 0.83	38.9 ± 0.44	38.0 ± 0.24	0.32	0.265
4 h post-feeding	38.3 ± 0.43	39.4 ± 0.29	38.9 ± 0.60	0.26	0.068
Mean	38.3 ± 0.54	39.2 ± 0.12	38.3 ± 0.23	0.22	0.209
**pH**					
0 h post-feeding	7.3 ± 0.11	7.4 ± 0.05	7.3 ± 0.04	0.04	0.314
4 h post-feeding	6.7 ± 0.13	6.6 ± 0.15	6.9 ± 0.30	0.10	0.660
Mean	7.0 ± 0.12	6.9 ± 0.09	7.1 ± 0.11	0.06	0.825
**BUN, mg/dL**					
0 h post-feeding	13.5 ± 1.77	14.5 ± 1.36	14.6 ± 2.06	0.94	0.904
4 h post-feeding	15.0 ± 1.37ᵇ	21.2 ± 1.89ᵃ	18.8 ± 1.96ᵃᵇ	1.14	0.047
Mean	14.3 ± 1.50	17.8 ± 1.58	16.7 ± 1.97	0.98	0.387
**NH₃–N, mg%**					
0 h post-feeding	10.70 ± 0.45	10.42 ± 0.43	10.76 ± 0.44	0.24	0.790
4 h post-feeding	12.75 ± 0.30ᵇ	14.24 ± 0.35ᵃ	13.17 ± 0.41ᵇ	0.25	0.001
Mean	11.72 ± 0.22	12.33 ± 0.23	12.12 ± 0.26	0.14	0.137
**Volatile fatty acids, mM**					
**Acetate**					
0 h post-feeding	78.61 ± 1.40	74.94 ± 2.06	74.69 ± 1.52	1.03	0.305
4 h post-feeding	88.27 ± 1.17	87.23 ± 1.37	86.19 ± 2.51	0.88	0.747
Mean	83.44 ± 1.22	81.09 ± 1.63	80.14 ± 1.63	0.88	0.407
**Propionate**					
0 h post-feeding	25.91 ± 1.82	19.84 ± 1.59	20.31 ± 1.95	1.19	0.062
4 h post-feeding	28.91 ± 2.17	24.88 ± 0.22	24.33 ± 1.26	0.98	0.089
Mean	27.41 ± 1.89ᵃ	22.36 ± 0.77ᵇ	22.23 ± 1.67ᵇ	1.03	0.044
**Butyrate**					
0 h post-feeding	7.87 ± 0.56ᵇ	8.86 ± 0.71ᵃ	7.74 ± 0.49ᵇ	0.35	0.013
4 h post-feeding	11.81 ± 1.09	10.76 ± 0.62	9.52 ± 1.05	0.55	0.322
Mean	9.84 ± 0.76	9.81 ± 0.46	8.82 ± 0.59	0.36	0.320

ᵃ,ᵇMeans within the same row with different superscripts differ significantly (p < 0.05).

Values are expressed as mean ± SEM.

SEM = standard error of the mean; TMFc = ensiled total mixed fiber with cassava pulp; TMFe = ensiled total mixed fiber with cassava bioethanol waste; BUN = blood urea nitrogen; NH₃–N = ammonia nitrogen.

### Milk yield and composition

Milk production and composition data are summarized in Table 4. Although the difference was not statistically significant, cows fed ensiled TMFc tended to produce higher daily milk yield (15.5 ± 0.82 kg/day; p = 0.069). This tendency may indicate differences in nutrient partitioning and energy utilization between cassava-derived byproducts. Milk composition, including fat, protein, lactose, solids-not-fat, and total solids, was not significantly affected by dietary treatment (p > 0.05). Milk fat content averaged 3.51 ± 0.37%, 3.51 ± 0.25%, and 3.57 ± 0.34% in the guinea grass, ensiled TMFc, and ensiled TMFe groups, respectively, whereas milk protein content ranged from 3.06 ± 0.07% to 3.14 ± 0.04%.

## DISCUSSION

### Effects of ensiled TMF on BW, feed intake, and nutrient digestibility

This study provides original comparative evidence on the use of ensiled TMF formulated from two distinct cassava-derived agro-industrial byproducts, cassava pulp and cassava bioethanol waste, as alternative roughage sources for early-lactating dairy cows. No significant differences were observed in initial BW, final BW, or ADG among the dietary treatments (p > 0.05). These findings indicate that replacing guinea grass with ensiled TMFc or ensiled TMFe did not influence body weight change during early lactation under the conditions of the present study. Although previous studies have reported improvements in growth performance following the inclusion of cassava-derived byproducts in ruminant diets [32], such responses were not observed in the present experiment. The absence of significant differences may be attributed to biological variation among animals, the relatively small experimental population, and the limited duration of the feeding trial. Therefore, no conclusion regarding enhanced growth performance can be drawn from the present findings.

**Table 4:** Effects of ensiled total mixed fiber (TMF) on milk yield and milk composition in early-lactating dairy cows.

**Items**	**Guinea grass**	**TMFc**	**TMFe**	**SEM**	**p-value**
**Milk production, kg/day**					
Milk yield	13.6 ± 0.83	15.5 ± 0.82	14.4 ± 0.84	0.97	0.069
4% FCM	13.1 ± 0.79	15.2 ± 0.79	14.1 ± 0.79	0.85	0.214
**Milk composition, %**					
Fat	3.51 ± 0.37	3.51 ± 0.25	3.57 ± 0.34	0.17	0.976
Protein	3.14 ± 0.04	3.13 ± 0.05	3.06 ± 0.07	0.03	0.510
Lactose	4.68 ± 0.06	4.55 ± 0.08	4.53 ± 0.09	0.05	0.133
Solids-not-fat	8.57 ± 0.15	8.39 ± 0.11	8.30 ± 0.17	0.08	0.211
Total solids	12.08 ± 0.33	11.90 ± 0.28	11.87 ± 0.44	0.20	0.827

FCM = fat-corrected milk. 4% FCM = 0.432 × (kg of milk) + 15 × (kg of fat). Values are expressed as mean ± SEM. SEM = standard error of the mean; TMFc = ensiled total mixed fiber with cassava pulp; TMFe = ensiled total mixed fiber with cassava bioethanol waste.

The greater DMI observed in cows fed the ensiled TMFe diet was noteworthy because this diet contained relatively higher fiber and lignin concentrations than the other roughage sources. This response may be associated with favorable physical and sensory characteristics of the ensiled feed, including moisture content, texture, and fermentation aroma, which likely enhanced palatability and voluntary feed intake. Furthermore, the inclusion of cassava bioethanol waste together with the ensiling process may have modified the physical characteristics of the fiber matrix, thereby reducing the physical limitations normally associated with fibrous feeds without inducing excessive rumen fill or satiety. Consequently, cows receiving the TMFe diet achieved significantly greater nutrient and ME intakes despite consuming a diet with higher NDF and ADF concentrations. These findings agree with previous studies demonstrating that cassava-derived byproducts can be successfully incorporated into dairy cow diets to improve feed intake while maintaining rumen fermentation and nutrient utilization [33, 34]. Likewise, ME intake was significantly greater in cows fed ensiled TMFe than in those fed ensiled TMFc or guinea grass, reflecting both greater voluntary feed intake and increased energy supply. These findings support previous reports indicating that cassava bioethanol waste provides a highly fermentable energy source suitable for ruminant feeding [21, 35]. Collectively, the present results suggest that ensiled TMFe represents a practical strategy for increasing nutrient intake and energy supply in early-lactating dairy cows.

Although nutrient intake differed significantly among dietary treatments, apparent digestibility of DM, OM, CP, NDF, and ADF remained unaffected (p > 0.05). These findings demonstrate that the inclusion of ensiled TMF, including TMFe with its relatively greater fiber and lignin contents, did not compromise digestive efficiency in early-lactating dairy cows. The increased DM and ME intakes observed in cows fed TMFe were therefore achieved without adversely affecting nutrient digestion. This response may reflect the capacity of the rumen microbial ecosystem to adapt to diets containing fibrous agro-industrial byproducts while maintaining normal digestive activity. Furthermore, although feed intake increased, the increase was apparently insufficient to markedly reduce ruminal retention time or impair microbial degradation of dietary nutrients. These observations are consistent with previous reports showing that appropriately processed cassava-derived byproducts can be incorporated into ruminant diets without negatively affecting nutrient digestibility [21, 33]. Overall, the present findings indicate that ensiled TMF can improve nutrient and energy intake while maintaining digestive efficiency, supporting its suitability as an alternative roughage source for lactating dairy cows.

### Effects of ensiled TMF on rumen fermentation characteristics and blood metabolites

Ruminal temperature and pH remained within normal physiological ranges throughout the experiment and were not significantly influenced by dietary treatment (p > 0.05). Mean ruminal pH remained within the range considered optimal for microbial fermentation (approximately 6.5–7.0), indicating that replacing guinea grass with ensiled TMFc or ensiled TMFe did not adversely affect rumen stability or predispose cows to subacute ruminal acidosis. These findings further suggest that the inclusion of cassava-derived agro-industrial by-products as primary roughage sources can maintain a stable ruminal environment during early lactation. Similar observations have been reported previously, in which cassava bioethanol waste, including yeast-fermented products, maintained normal ruminal pH and supported stable fermentation patterns [33].

The significantly greater BUN and ruminal NH₃–N concentrations observed in cows fed the ensiled TMFc diet indicate increased ruminal nitrogen availability and greater degradation of dietary nitrogen. This response most likely reflects rapid urea hydrolysis combined with the presence of readily fermentable carbohydrates supplied by cassava pulp. Urea is rapidly converted to ammonia within the rumen, and when synchronized with an adequate supply of fermentable carbohydrates, ruminal microorganisms can efficiently utilize the released ammonia for microbial growth [36, 37]. However, elevated BUN concentrations may also indicate that nitrogen supply exceeded the availability of fermentable energy, resulting in excess ammonia absorption and subsequent conversion to urea in the liver before excretion [38]. These findings emphasize the importance of synchronizing ruminal energy and nitrogen availability when formulating TMF-based diets. Similar responses have been reported in urea-supplemented cassava diets, in which ruminal nitrogen metabolism was influenced by the balance between degradable nitrogen and fermentable carbohydrate supply [39]. Therefore, although the ensiled TMFc diet enhanced ruminal nitrogen release, optimizing the energy-to-nitrogen ratio may further improve nitrogen utilization efficiency and reduce unnecessary urea formation. Because microbial protein synthesis, microbial population dynamics, and whole-animal nitrogen balance were not determined in the present study, the efficiency with which the additional ruminal ammonia was incorporated into microbial protein could not be directly evaluated.

Differences in VFA profiles among dietary treatments further indicate that the type of cassava-derived by-product influenced ruminal fermentation pathways. The greater mean propionate concentration observed in cows fed guinea grass suggests a shift toward glucogenic fermentation compared with the ensiled TMF diets. These differences were likely associated with variation in carbohydrate composition and fermentation characteristics among the roughage sources. Diets containing ensiled TMF, which included substantial proportions of bagasse and cassava bioethanol waste, may have favored acetate-producing fermentation pathways because of their greater structural carbohydrate content. Acetate is the principal precursor for milk fat synthesis, whereas propionate serves as the major glucogenic precursor for hepatic glucose production and is therefore closely associated with lactose synthesis, energy metabolism, and milk production [40]. The relatively lower propionate concentrations observed in cows fed ensiled TMF may therefore reflect reduced starch availability together with increased fermentation of structural carbohydrates, resulting in a greater acetate-to-propionate ratio.

Previous studies have demonstrated that fermentation of cassava pulp with urea and molasses can increase ruminal propionate production and improve energy utilization [36]. In contrast, butyrate primarily functions as an important energy source for ruminal epithelial tissues and contributes to epithelial development and function. Although significant differences were observed in propionate and butyrate concentrations among treatments, these changes were not accompanied by corresponding differences in milk yield or milk composition. This finding suggests that the observed alterations in ruminal fermentation patterns were insufficient to produce measurable changes in animal performance under the conditions of the present study. Consequently, further optimization of the roughage-to-concentrate ratio and synchronization of carbohydrate fermentation characteristics may further improve VFA production patterns and enhance energy utilization in high-producing dairy cows.

### Effects of ensiled TMF on milk yield and milk composition

Although milk yield was not significantly affected by dietary treatment, cows fed the ensiled TMFc diet showed a tendency toward greater milk production than those fed ensiled TMFe or guinea grass (p = 0.069). Although this tendency did not reach statistical significance, it suggests that ensiled TMFc may provide a nutritional environment that is more favorable for milk synthesis. This response may be attributed to the greater availability of rapidly fermentable carbohydrates from cassava pulp together with degradable nitrogen supplied by urea, which may have improved ruminal nutrient availability and microbial activity [36]. Similar responses have been reported previously, in which diets containing urea-treated cassava pulp enhanced rumen fermentation and were associated with improved milk production in dairy cows [32]. However, the mechanisms responsible for the tendency toward greater milk yield in the present study cannot be definitively established because microbial protein synthesis, rumen microbial populations, and metabolic indicators such as blood glucose, insulin, and VFA absorption were not evaluated. Consequently, although the observed response was likely associated with differences in nutrient availability and ruminal fermentation, further investigations are required to confirm the underlying physiological mechanisms.

The milk yields obtained in the present study were lower than those commonly reported for high-producing Holstein cows managed under temperate production systems. This difference is likely attributable to the use of Holstein Friesian crossbred cows raised under tropical environmental conditions in Thailand, where heat stress, forage quality, management practices, and genetic background may limit milk production potential. Therefore, the production levels observed in the present study are representative of practical dairy production systems in tropical regions and provide realistic information regarding the utilization of cassava-derived roughage resources under field conditions.

Milk composition, including fat, protein, lactose, solids-not-fat, and total solids, was not significantly affected by dietary treatment (p > 0.05). These findings indicate that replacing guinea grass with ensiled TMFc or ensiled TMFe did not adversely affect mammary nutrient partitioning or milk synthesis. The maintenance of normal milk composition despite differences in feed intake and ruminal fermentation suggests that both cassava-derived roughage sources adequately supplied nutrients required for milk component synthesis. These observations are consistent with previous studies demonstrating that cassava pulp supplementation maintains stable milk composition, particularly lactose concentration, while occasionally improving milk protein and fat depending on dietary formulation [41]. Similarly, diets containing yeast-fermented cassava bioethanol waste have been reported to support milk protein synthesis without altering milk fat or lactose concentrations because of their relatively high CP content and favorable fermentation characteristics [42]. Collectively, these findings demonstrate that both cassava pulp and cassava bioethanol waste can be successfully incorporated into ensiled TMF without compromising milk quality, thereby supporting their suitability as alternative roughage resources for lactating dairy cows.

### Practical implications and study significance

The present findings provide novel comparative evidence regarding the utilization of two distinct cassava-derived agro-industrial byproducts as primary roughage sources within ensiled TMF systems for early-lactating dairy cows. Unlike previous investigations that primarily evaluated cassava byproducts as concentrate ingredients, protein supplements, or partial feed additives, the present study demonstrated their effectiveness as complete roughage replacements within an ensiled feeding system. Specifically, the results showed that TMFe increased voluntary feed intake and ME intake despite containing greater fiber and lignin concentrations, whereas TMFc enhanced ruminal nitrogen availability through increased NH₃–N and BUN concentrations without adversely affecting ruminal pH, nutrient digestibility, or milk composition.

These findings also have important practical implications for sustainable dairy production in tropical regions. Cassava pulp and cassava bioethanol waste are abundantly available agro-industrial residues that are frequently underutilized or disposed of as waste. Their successful incorporation into ensiled TMF provides an opportunity to convert low-value industrial byproducts into nutritionally valuable livestock feed while reducing dependence on conventional roughage resources such as guinea grass and rice straw, whose availability and quality often fluctuate seasonally. Consequently, adopting cassava-based ensiled TMF may help reduce feed shortages, lower feeding costs, improve resource-use efficiency, and support circular bioeconomy strategies by valorizing cassava-processing residues.

### Study limitations and future perspectives

Several limitations should be considered when interpreting the findings of the present study. First, the number of animals per treatment was relatively small (n = 6), which may have limited the statistical power to detect treatment effects for variables exhibiting only tendency-level significance. Therefore, the results should be interpreted with appropriate caution, particularly for milk yield and other parameters approaching statistical significance.

Second, although the ensiled TMF appeared well preserved based on the absence of visible mold, abnormal odor, and spoilage, the ensiling period was limited to 7 days, and silage fermentation characteristics, including silage pH, lactic acid, acetic acid, butyric acid, and NH₃–N concentrations, were not determined. Consequently, silage quality could only be evaluated through physical observations rather than comprehensive fermentation analyses. Future studies should include detailed silage fermentation assessments to better characterize the preservation efficiency and nutritive quality of cassava-based ensiled TMF.

Third, important indicators of rumen function and dietary fiber effectiveness were not evaluated. Measurements of peNDF, particle size distribution, chewing and rumination activities, total VFA concentration, and the acetate-to-propionate ratio were unavailable. Consequently, the effects of dietary treatments on ruminal fermentation dynamics, energy partitioning, and the physical effectiveness of dietary fiber could not be comprehensively assessed.

In addition, residual hydrogen cyanide (HCN) concentrations in the ensiled diets and blood thiocyanate concentrations in the cows were not determined. Although no clinical signs of toxicity were observed during the experimental period, the absence of these measurements precluded a comprehensive evaluation of the safety of cassava-derived byproducts. Future investigations should incorporate chemical analyses of cyanogenic compounds together with physiological biomarkers to confirm the safety of long-term feeding of cassava-based ensiled TMF.

Furthermore, microbial protein synthesis, urinary purine derivative excretion, nitrogen balance, rumen microbial populations, and metabolic indicators, including blood glucose and insulin concentrations, were not evaluated. Consequently, the mechanisms underlying nitrogen utilization, microbial efficiency, and energy metabolism could not be fully elucidated. Additional studies integrating microbial, metabolic, and molecular approaches would provide a more comprehensive understanding of the biological responses to cassava-derived ensiled TMF.

The present study also did not evaluate enteric methane emissions or other environmental indicators associated with ruminal fermentation. Therefore, the potential contribution of cassava-based ensiled TMF to reducing greenhouse gas emissions and improving environmental sustainability remains unclear. Likewise, economic evaluations, including feed cost, feed efficiency economics, milk revenue, and income over feed cost, were beyond the scope of the present study. These analyses are essential for determining the practical feasibility and economic benefits of incorporating cassava-derived ensiled TMF into commercial dairy production systems.

Finally, environmental variables associated with tropical production systems, including ambient temperature, relative humidity, temperature–humidity index, and seasonal variation, were not monitored throughout the experiment. Consequently, the potential influence of heat stress and seasonal environmental conditions on feed intake, rumen fermentation, nutrient utilization, and milk production could not be separated from dietary effects.

Future studies should therefore include larger experimental populations, longer feeding periods, comprehensive silage fermentation analyses, peNDF determination, rumen microbial characterization, microbial protein synthesis, nitrogen utilization, metabolic and endocrine profiling, HCN safety evaluations, methane emission measurements, economic assessments, and environmental monitoring. Such investigations will provide a more comprehensive understanding of the nutritional, physiological, economic, environmental, and sustainability implications of utilizing cassava pulp- and cassava bioethanol waste-based ensiled TMF as alternative roughage sources for dairy production systems.

## CONCLUSION

The present study demonstrated that ensiled TMF formulated from cassava-derived agro-industrial byproducts can successfully replace guinea grass as a roughage source in early-lactating dairy cows without adversely affecting body weight, nutrient digestibility, rumen stability, or milk composition. Cows fed the TMFe diet exhibited significantly greater DMI, nutrient intake, and ME intake than those fed guinea grass or TMFc, whereas apparent digestibility of DM, OM, CP, NDF, and ADF remained unchanged among treatments. The TMFc diet enhanced ruminal nitrogen metabolism, as evidenced by higher NH₃–N and BUN concentrations, and showed a tendency toward greater milk yield, although this response did not reach statistical significance. Despite differences in ruminal fermentation characteristics, ruminal pH remained within the physiological range and milk composition was maintained across all treatments, indicating that both cassava-derived roughage sources supported normal rumen function and lactational performance.

These findings demonstrate the practical potential of ensiled TMFc and TMFe as sustainable alternative roughage resources for dairy production in tropical regions. The utilization of cassava pulp and cassava bioethanol waste not only provides nutritionally suitable feed ingredients but also promotes the valorization of agro-industrial residues, thereby contributing to circular bioeconomy strategies, reducing waste disposal, and decreasing reliance on conventional forage resources that are often limited in availability and quality.

A major strength of this study is that it provides the first direct comparison of ensiled TMFc and ensiled TMFe as primary roughage sources in early-lactating dairy cows, thereby extending current knowledge beyond previous studies that primarily evaluated cassava-derived byproducts as concentrate ingredients or feed supplements. However, the absence of detailed silage fermentation characteristics, peNDF determination, microbial protein synthesis, rumen microbial analyses, metabolic profiling, safety evaluation of residual HCN, economic assessment, and environmental measurements limits a comprehensive understanding of the mechanisms underlying the observed responses.

Overall, ensiled TMF prepared from cassava pulp or cassava bioethanol waste represents a promising, nutritionally effective, and environmentally sustainable alternative roughage source for early-lactating dairy cows. Among the two formulations evaluated, TMFe was more effective in enhancing feed and energy intake, whereas TMFc promoted greater ruminal nitrogen availability and showed a tendency toward higher milk yield, highlighting the complementary nutritional characteristics of these two cassava-derived agro-industrial byproducts for tropical dairy production systems.

## DATA AVAILABILITY

The datasets generated and/or analyzed during the current study can be available from the corresponding author upon reasonable request.

## GENERATIVE AI DECLARATION

The authors declare that generative artificial intelligence (AI) tools were used solely to improve language, grammar, and readability during manuscript preparation. All scientific content, data analysis, interpretation of results, and conclusions were developed and verified by the authors. The authors take full responsibility for the accuracy, integrity, and originality of the work presented, and no AI tool was listed as an author. 

## AUTHORS’ CONTRIBUTIONS

PK, ST, and WL: Conceived and designed the study, supervised the overall project, interpreted the findings, and critically revised the manuscript. NC: Conducted the experiments, collected the samples, performed laboratory analyses, managed data collection and statistical analysis, and drafted the manuscript. All authors have read and approved the final version of the manuscript.
